# Gender Representation Among Editors of Major Pediatric Journals

**DOI:** 10.1001/jamanetworkopen.2023.21533

**Published:** 2023-07-03

**Authors:** Jessica M. Allan, Jeremy W. Jacobs, Nancy D. Spector, Priscila Rodrigues Armijo, Garrett S. Booth, Julie K. Silver

**Affiliations:** 1 Department of Pediatrics, Palo Alto Medical Foundation, Stanford University School of Medicine, Palo Alto, California; 2 Department of Laboratory Medicine, Yale School of Medicine, New Haven, Connecticut; 3 Executive Leadership in Academic Medicine, Department of Pediatrics, Drexel University College of Medicine, Drexel University, Philadelphia, Pennsylvania; 4 Academic Affairs, Department of Surgery, University of Nebraska Medical Center, Omaha; 5 Department of Pathology, Microbiology, and Immunology, Vanderbilt University Medical Center, Nashville, Tennessee; 6 Department of Physical Medicine and Rehabilitation, Harvard Medical School, Boston, Massachusetts

## Abstract

This cross-sectional study analyzes inequities in the gender of editors for 3 major pediatric journals.

## Introduction

Gender equity among medical journal editors is a longstanding, remediable issue. In 2016, Fishman et al^1^ examined gender equity among editors of 3 major pediatrics journals (*Journal of Pediatrics*, *JAMA Pediatrics*, and *Pediatrics*) and found increasing representation of female editors from 2001 (17.8% [16 of 90]) to 2016 (39.8% [41 of 103]); nevertheless, their overall inclusion was significantly less than specialty benchmarks.

Editorial roles confer numerous career benefits and are frequently part of rank promotion criteria. Given the lack of progress for women at higher levels of promotion,^2^ each component deserves scrutiny.

Seven years after the study by Fishman et al,^1^ we conducted a reanalysis to investigate whether inequities persist. We hypothesized that despite some progress, inequities would still remain.

## Methods

This cross-sectional study analyzed editor gender annually from 2017 to 2023 for the *Journal of Pediatrics*, *JAMA Pediatrics*, and *Pediatrics* via masthead analysis. Perceived gender was classified as woman, man, or other via review of online pronouns or photographs. No individuals were perceived to identify as “other,” so we report gender as woman or man. Gender was coded independently by 3 authors (J.M.A., J.W.J., and P.R.A.; 100% concordance).

The proportion of women editors overall and the proportion of physician editors were assessed against 2 benchmarks: parity (50%) and equity (proportion of medical school faculty in pediatrics and pediatric physician medical school faculty using Association of American Medical Colleges data, reported as male or female per the source). This study followed the Strengthening the Reporting of Observational Studies in Epidemiology (STROBE) reporting guideline and did not require ethics approval as all data are publicly available. Descriptive statistics were performed using PRISM, version 9.5.1 (GraphPad). All *P* values were from 2-sided tests and results were deemed statistically significant at *P* < .05.

## Results

Between 2017 and 2023, 773 total (722 physician) editor positions were analyzed. Among all editors, 39.2% (303 of 773) were women, and 38.4% of physician editors (277 of 722) were women. Compared with equity for the most recent year (2023), women overall were significantly underrepresented in editor positions for *Journal of Pediatrics* (−24.4%), and women physicians were significantly underrepresented in editor positions for *Journal of Pediatrics* (−20.9%) and *Pediatrics* (−20.2%) (Table; Figure). The most improvement among both groups was seen for *JAMA Pediatrics,* the only journal to achieve parity for both groups.

**Table.** Analysis of the Proportion of All Women Editors and Women Physician Editors Compared With Equity Benchmarks From 2017 to 2023

Year	*Journal of Pediatrics*					*JAMA Pediatrics*					*Pediatrics*				
	No./total No. (%)	95% CI, %	Difference from equity, %	*P* value	No./total No. (%)		95% CI, %	Difference from equity, %	*P* value	No./total No. (%)		95% CI, %	Difference from equity, %	*P* value
**All women editors**															
2017^a^	11/38 (29.0)	17.0-44.8	−28.4	<.001	7/21 (33.3)		17.2-54.6	−24.1	.03	15/39 (38.5)		24.9-54.1	−18.9	.02
2018^a^	11/36 (30.6)	18.0-46.9	−26.8	.001	10/24 (41.7)		24.5-61.2	−15.7	.15	14/41 (34.2)		21.6-49.5	−23.2	.004
2019^a^	15/41 (36.6)	23.6-51.9	−20.8	.01	9/22 (40.9)		23.3-61.3	−16.5	.13	14/41 (34.2)		21.6-49.5	−23.2	.004
2020^b^	15/43 (34.9)	22.4-49.8	−25.4	<.001	10/22 (45.5)		26.9-65.3	−14.8	.19	17/44 (38.6)		25.7-53.4	−21.7	.005
2021^c^	16/47 (34.0)	22.2-48.3	−26.9	<.001	10/22 (45.5)		26.9-65.3	−15.4	.19	22/47 (46.8)		33.3-60.8	−14.1	.05
2022^d^	18/49 (36.7)	24.7-50.7	−24.4	<.001	13/25 (52.0)		33.5-70.0	−9.1	.41	23/50 (46.0)		33.0-59.6	−15.1	.03
2023^d^	18/49 (36.7)	24.7-50.7	−24.4	<.001	13/25 (52.0)		33.5-70.0	−9.1	.41	22/47 (46.8)		33.3-60.8	−14.3	.05
**Women physician editors**															
2017^a^	11/37 (29.7)	17.5-45.8	−27.7	.001	6/18 (33.3)		16.3-56.3	−24.1	.06	15/39 (38.5)		24.9-54.1	−18.9	.02
2018^a^	11/35 (31.4)	18.6-48.0	−26.0	.003	9/21 (42.9)		24.5-63.5	−14.5	.19	14/41 (34.2)		21.6-49.5	−23.3	.004
2019^a^	15/40 (37.5)	24.2-53.0	−19.9	.02	8/19 (42.1)		23.1-63.7	−15.3	.25	14/41 (34.2)		21.6-49.5	−23.3	.004
2020^e^	15/41 (36.6)	23.6-51.9	−22.0	.006	9/19 (47.4)		27.3-68.3	−11.2	.36	17/44 (38.6)		25.7-53.4	−20.0	.009
2021^f^	16/45 (35.6)	23.2-50.2	−23.5	.002	9/19 (47.4)		27.3-68.3	−11.7	.35	17/42 (40.5)		27.0-55.5	−18.6	.02
2022^g^	18/47 (38.3)	25.8-52.6	−20.9	.005	11/21 (52.4)		32.4-71.7	−6.8	.66	17/44 (38.6)		25.7-53.4	−20.6	.008
2023^g^	18/47 (38.3)	25.8-52.6	−20.9	.005	11/21 (52.4)		32.4-71.7	−6.8	.66	16/41 (39.0)		25.7-54.3	−20.2	.01

^a^ Benchmarks: Percentage of women physicians among US medical school pediatric physician faculty in 2017.^3^

^b^ Percentage of women (physicians and nonphysicians) among US medical school pediatric faculty (physicians and nonphysicians) in 2020. From: AAMC. Table 14: US medical school faculty by gender, degree, and department, 2020. Accessed March 15, 2023 (https://www.aamc.org/media/8871/download?attachment).

^c^ Percentage of women (physicians and nonphysicians) among US medical school pediatric faculty (physicians and nonphysicians) in 2021. From: AAMC. Table 14: US medical school faculty by gender, degree, and department, 2021. Accessed March 15, 2023 (https://www.aamc.org/media/9741/download?attachment).

^d^ Percentage of women (physicians and nonphysicians) among US medical school pediatric faculty (physicians and nonphysicians) in 2022. From: AAMC. Table 14: US medical school faculty by gender, degree, and department, 2022. Accessed March 15, 2023 (https://www.aamc.org/media/8446/download?attachment).

^e^ Percentage of women physicians among US medical school pediatric physician faculty in 2020. From: AAMC. Table 14: US medical school faculty by gender, degree, and department, 2020. Accessed March 15, 2023 (https://www.aamc.org/media/8871/download?attachment).

^f^ Percentage of women physicians among US medical school pediatric physician faculty in 2021. From: AAMC. Table 14: US medical school faculty by gender, degree, and department, 2021. Accessed March 15, 2023 (https://www.aamc.org/media/9741/download?attachment).

^g^ Percentage of women physicians among US medical school pediatric physician faculty in 2022. From: AAMC. Table 14: US medical school faculty by gender, degree, and department, 2022. Accessed March 15, 2023 (https://www.aamc.org/media/8446/download?attachment).

**Figure.**
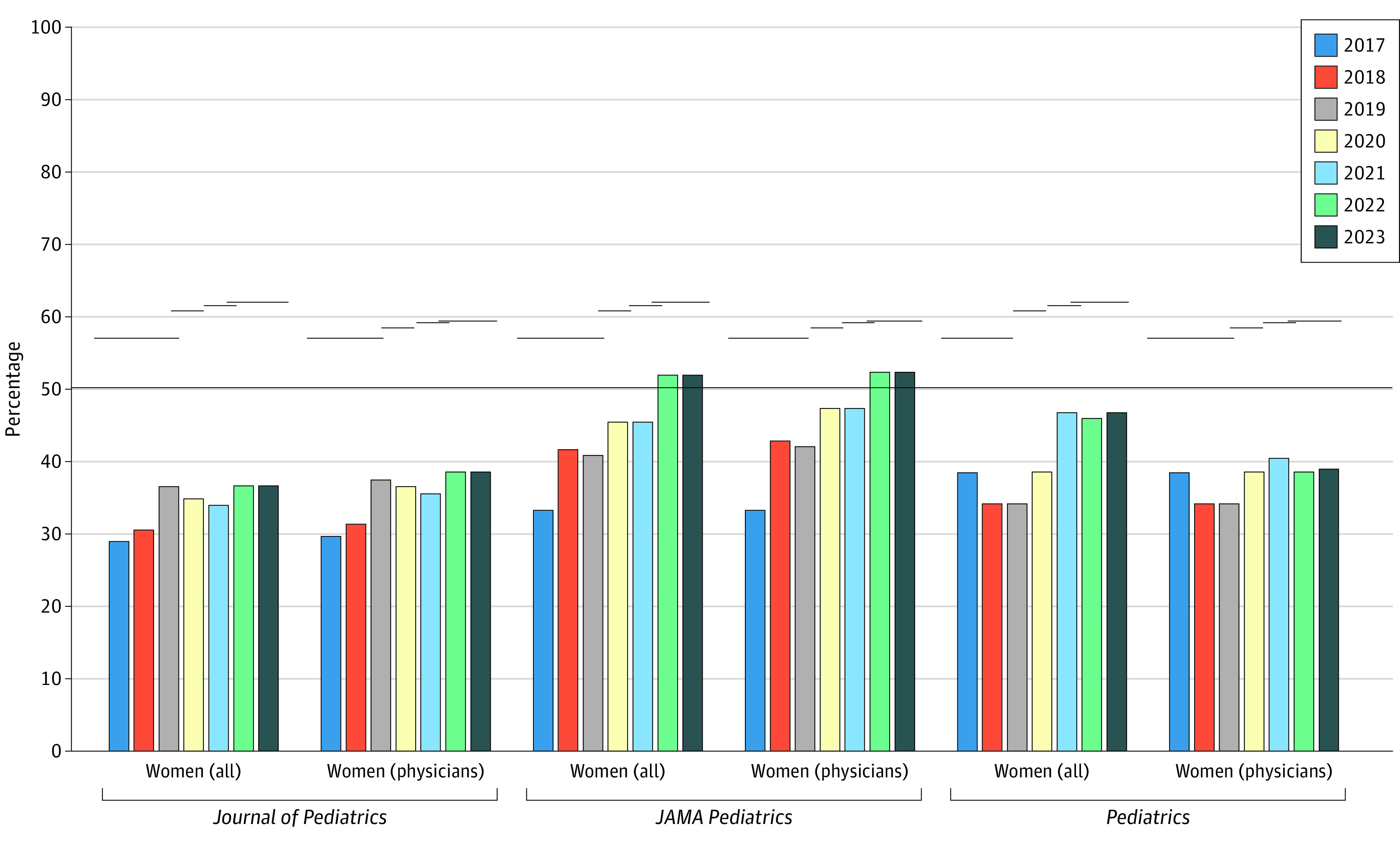
Women Editors in Pediatric Journals (2017-2023) Annual percentage of women and women physicians among all editors and physician editors, respectively, compared with parity and equity. The long solid horizontal line at 50% indicates parity (50:50 gender balance). The shorter solid horizontal lines indicate equity (proportion of all women pediatric medical faculty and women physician pediatric medical faculty) using Association of American Medical Colleges data.

## Discussion

During the study period, *Journal of Pediatrics* and *Pediatrics* were below all benchmarks (Table; Figure). *JAMA Pediatrics* was below all specialty benchmarks for all years but was above parity for the past 2 years. Continued underrepresentation of women editors in pediatric journals is concerning because this is a well-documented inequity. Amrein et al^4^ showed that *Journal of Pediatrics* and *Pediatrics* (*JAMA Pediatrics* was not included) experienced progress compared with prior reports in 2005 and 2011; however, the percentages of women editors remained low compared with their high representation in the specialty. Fishman and colleagues^1^ found increased representation of women editors of the 3 journals from 17.8% (16 of 90) in 2001 to 39.8% (41 of 103) in 2016. Our reanalysis shows continued improvement for women editors, from 33.7% (33 of 98) in 2017 to 43.8% (53 of 121) in 2023—yet they remain below parity and equity benchmarks.

We did not study causality, and the reasons for underrepresentation of women on pediatric journal editorial boards are likely multifactorial. For example, implicit (unconscious) bias and the underrepresentation of women as senior authors may be contributing factors.^1,3^

Medical societies that own or are affiliated with journals should be accountable for persistent inequities among editors. Societies that own journals should adhere to owner obligations that include ensuring that editors behave in a manner that is compatible with public trust.^5^ Societies that support journals with persistent inequities for women editors are participating in interorganizational structural discrimination.^6^ Limitations of this study include reliance on online information and inability to account for the gender spectrum and other aspects of diversity (eg, race and ethnicity).

In summary, all pediatric journals studied demonstrated inequitable inclusion of women editors. This remediable issue should be addressed by both the journals and the affiliated medical societies.

## Notes

Supplement.
Data Sharing Statement
