# Supplementary material for: Gender Representation Among Editors of Major Pediatric Journals
Source: JAMA Netw Open. 2023 Jul 3;6(7):e2321533. doi: 10.1001/jamanetworkopen.2023.21533 (PMC10318475; doi:10.1001/jamanetworkopen.2023.21533)
Supplement: Supplement. — Data Sharing Statement [file jamanetwopen-e2321533-s001.pdf]

Data Sharing Statement

Allan. Gender Representation Among Editors of Major Pediatric Journals. *JAMA Netw Open*. Published July 03, 2023. doi:10.1001/jamanetworkopen.2023.21533

Data
Data available: Yes
Data types: Deidentified participant data
How to access data: Data available upon request from julie_silver@hms.harvard.edu
When available: With publication

Supporting Documents
Document types: None

Additional Information
Who can access the data: Researchers whose proposed use of the data has been approved
Types of analyses: For research
Mechanisms of data availability: With investigator support
